# Cognitive training for robotic surgery: a chance to optimize surgical training? A pilot study

**DOI:** 10.1007/s11701-020-01167-3

**Published:** 2020-11-13

**Authors:** Sandra Schönburg, Petra Anheuser, Jennifer Kranz, Paolo Fornara, Viktor Oubaid

**Affiliations:** 1 Department of Urology and Kidney Transplantation, Martin Luther University, Ernst-Grube-Straße 40, 06120 Halle (Saale), Germany; 2 Department of Urology, Asclepius Clinic Wandsbek, Alphonsstrasse 14, 22043 Hamburg, Germany; 3 Urology and Paediatric Urology, St. Antonius Hospital, Eschweiler, Germany; 4 German Aerospace Centre (DLR), Hamburg, Germany

**Keywords:** Robotic surgery, Surgical training, Simulator training, Cognitive training, Personal traits

## Abstract

The rapid rise of robotic-assisted surgery (RAS) has necessitated an efficient and standardized training curriculum. Cognitive training (CT) can significantly improve skills, such as attention, working memory and problem solving, and can enhance surgical capacity and support RAS training. This pilot study was carried out between 02/2019 and 04/2019. The participants included 33 student volunteers, randomized into 3 groups: group 1 received training using the da Vinci training simulator, group 2 received computer-based cognitive training, and group 3 was the control group without training. Before (T1) and after-training (T2), performance was measured. Additionally, expert ratings and self-evaluations were collected. Subjective evaluations of performance were supplemented by evaluations based on three scales from the revised NEO Personality Inventory (NEO PI-R). In total, 25 probands remained with complete data for further analyses: *n* = 8 (group 1), *n* = 7 (group 2) and *n* = 10 (group 3). There were no significant differences in T1 and T2 among all three groups. The average training gain of group 1 and 2 was 15.87% and 24.6%, respectively, (a restricting condition is the loss of the last training session in group 2). Analyses of semi-structured psychological interviews (SPIs) revealed no significant differences for T1, but in T2, significance occurred at ‘self-reflection’ for group 2 (*F*(2.22) = 8.56; *p* < .005). The efficacy of CT in training highly complex and difficult procedures, such as RAS, is a proven and accepted fact. Further investigation involving higher numbers of training trials (while also being cost effective) should be performed.

## Background

The learning process of operative skills is traditionally based on surgical training, in which the trainee, under the supervision of an experienced surgeon, performs certain operative steps, usually directly on the patient. Traditional surgical training is a time consuming and costly process of varying effectiveness with a distinct learning curve. With the rapid growth of modern minimally invasive methods and increased concerns and standards regarding patient safety and workload, this procedure is becoming an anachronism. Consequently, simulators have been included in surgical training and have been demonstrated to reduce the time spent in achieving basic skills [1,2], but simulators and virtual trainers remain limited and expensive [3–6].

Additional teaching strategies of learning processes are predominantly known from flight training and the sport sector, but these strategies are in musical form and based on neurophysiological and motoric correlates [7–10]. This well-recognized and validated tool of cognitive training (CT) has for years been implemented and successfully used in different forms, such as mental rehearsal but also as cognitive task analysis (CTA) or computer-based cognitive simulation of pilots and athletes [11]. Additionally, cognitive training correlates with increased attentional stability and self-confidence [12, 13].

Understanding of the relationship between cognitive skills and performance in medical, especially surgical, education has increased in recent years and has originated approaches for implementing minimally invasive surgical training. However, CT is not yet validated and implemented in surgical education. The purpose of our study is to verify the importance of cognitive training in learning robotic basics.

## Material and methods

This pilot study was carried out prospectively and randomized by lot procedures between February 2019 and April 2019. The test plan was prepared in cooperation with the German Aerospace Centre (“Deutsches Zentrum für Luft- und Raumfahrt”, DLR, Hamburg). The participants in the study were 33 student volunteers. In accordance with legal requirements, each participant was provided detailed information and gave informed consent before the study began. All participants were first questioned about their performance as part of a semi-structured psychological interview (SPI). The SPI includes assessments of the participants’ skills to accurately self-assess performance and communicate experience. For this purpose, an interview guide was developed, and an assessment system was established. The interview guide consists of defined questions, enables the addition of sub-questions, and was, therefore, more appropriate than a questionnaire:

- How pronounced was your concentration?
- How well did you achieve psychomotoric coordination?
- How much did you try?
- How do you rate your overall performance on the da Vinci simulator?
- Are you satisfied with the performance achieved?
- Would you change your course of action during a repetition?
- How effective was the training session (*F*or group 1 or group 2)?

The assessment was as follows: Participant responses were coded on a scale from 1 (not applicable) to 6 (completely true) on two dimensions. Dimension 1 was defined as “self-reflection” (the degree of accuracy of self-assessed performance), and dimension 2 was defined as “communication” (the extent to which the interviewee succeeds in describing himself/herself or his/her performance).

Furthermore, due to time constraints and ethical reasons, a reduced version of the Revised NEO Personality Inventory (NEO PI-R), published by Costa and McCrae, was used in this study. (Specifically, only the scales “Extraversion, Conscientiousness and Openness to experience” were applied.) [14] Our reduced version of the NEO PI-R was administered to all the participants. The NEO PI-R follows the so-called 5-factor model. This personality inventory is one of the most used personality questionnaires worldwide and has proven its psychometric qualities in thousands of scientific studies [15].

Subsequently, using computer-generated, simple randomization, the subjects were assigned to one of the three study groups purely by chance and absolutely independently of the subjects’ knowledge. The subjects were not preselected. The following study groups were distinguished and compared:

- Group 1: SIM – received training on the da Vinci simulator;
- Group 2: CT – received cognitive training;
- Group 3: received no training.

**Fig. 1:**
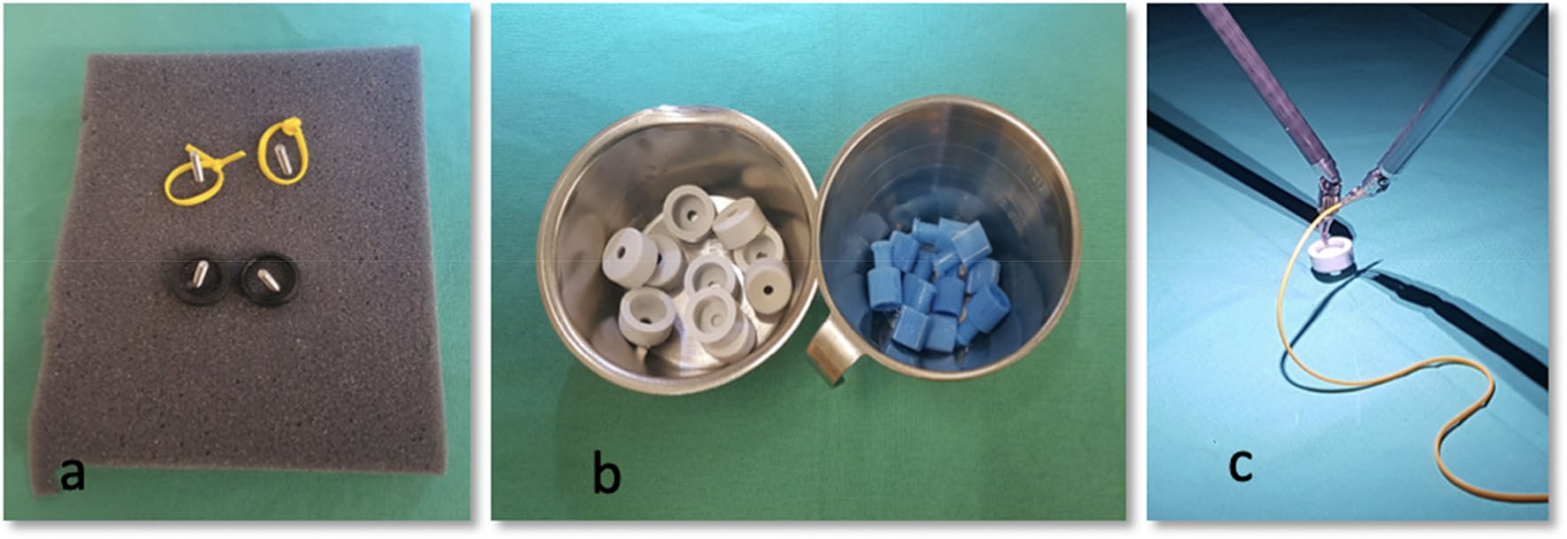
Exercises at the beginning: **a** Lift two yellow and two black rings onto the respective screw. Time was measured, **b** Second exercise at the beginning: Sort 10 blue and 10 grey connectors. Time was measured, **c** Thread a yellow rubber loop through a grey connector and tie and tighten the loop. Time was measured

After randomization, an initial examination on the da Vinci robot was performed by all participants; the examination, one of three exercises (see Fig. 1a−c) was repeated in an extended modified form after the completion of training or non-training. Modification was made to compensate for the memory effects of test 1.

**Fig. 2:**
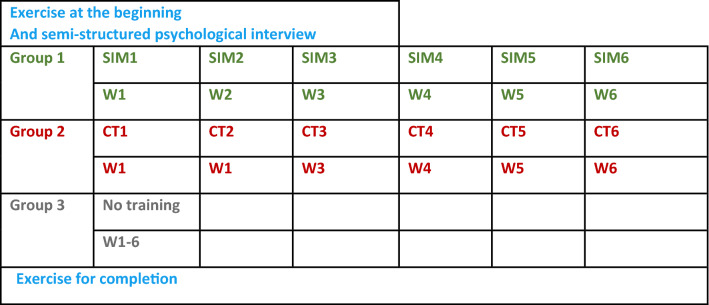
Flowsheet of the study: exercise at the beginning, training, and exercise for completion of the 3 groups (*CT* cognitive training, *W* week, *SIM* daVinci simulator)

**Fig. 3:**
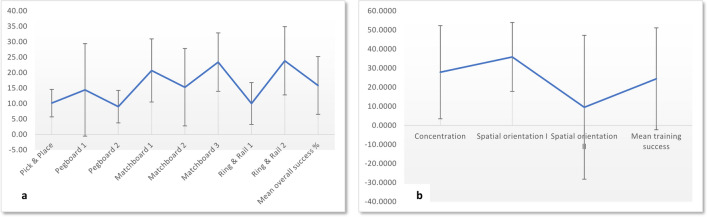
Training success: **a** Mean scores (%) and standard deviations for DaVinci training (*N* = 7, group 1) for the start/end of eight daVinci training modules and a composite percentage measure (mean overall success), **b** mean scores (%) for three training modules (group 2 members only) for training sessions 1 to 5 (*n* = 7)

During the training period between February 15, 2019, and April 12, 2019, the participants of group 1 passed through defined simulation modules on the da Vinci simulator. The following exercises were performed: EndoWrist manipulation 1 (each individual exercise lasted 15 min, and the first and last exercises were documented), EndoWrist manipulation 2 (each individual exercise involved three repetitions, and the first and last exercises were documented), repetition of EndoWrist manipulation 1, repetition of EndoWrist manipulation 2, needle control (each individual exercise lasted 10 min, and the first and last exercises were documented), needle driving (each individual exercise involved two repetitions, and both were documented). The total time spent in one run of all 6 training modules was 6 h per participant.

The participants of group 2 were given cognitive training tests on a personal computer (PC). The participants were informed as follows: These training modules were developed and validated [16] at the German Aerospace Centre (DLR) for use in the suitability diagnostics of pilots, air traffic controllers and European astronauts. These are the following modules: ental calculation, concentration and attention, spatial orientation, visual memory. The total time spent in one run of all six training modules was 6 h per participant. There was no training for the group 3 participants (see Fig. 2).

All subject data were recorded in a pseudonymized form while maintaining data protection. The collected data are described using descriptive statistical methods (exploratory) (Frequency distributions and statistical characteristics in tabular and/or graphical form) with *p* < 0.05 considered to be statistically significant. For statistical evaluation, the programs Microsoft® EXCEL 2010, Statistical Product and Service Solutions (SPSS) 22, and Prism 6 were used. The study was not carried out by the manufacturer.

## Results

After eliminating participants with missing data, *n* = 25 persons remained for further analyses, with *n* = 8 (group 1), *n* = 7 (group 2), and *n* = 10 (group 3).

In the basic performance evaluation (test 1), we found a significant difference between groups 1, 2 and 3. On average, the best result was achieved by the control group (group 3), and group 2 required the most time (see Table 1). In test 2, group 3 achieved the best result, and group 2 took the most time, as in test 1. However, in an indirect comparison, the greatest time reduction was presented group 1, which started from a higher time value in stest 1 and achieved a result comparable to that of group 3 in test 2.

**Table 1:** Mean scores and standard deviations for time consumed in tests T1 and T2 for three groups

	da Vinci task T1 [sec]		da Vinci task T2 [sec]	
	MW	SD	MW	SD
Group 1 (*n* = 8)	305.02	90.33	286.61	88.16
Group 2 (*n* = 7)	389.65	283.15	499.39	192.45
Group 3 (*n* = 10)	244.07	62.41	284.69	42.79

Mean scores and standard deviations for the exercises in test 1 (T1) and test 2 (T2) are displayed in Table 1. The ANOVA F statistic was significant in T2, exercise 3 (*F*(2.22) = 8.45; *p* = 0.002); the Scheffé procedure revealed a significant group difference between group 2 and group 1 or group 3.

The training success in group 1 was measured by calculating the difference between the first training score in the first training trial (“Start”) and the last training score in the last training trial (“End”). The average training gain for group 1 was 15.87% over all eight training measures (see Fig. 3).

The evaluation (ANOVA analysis) of personality characteristics using the NEO scales before test 1 and SPI measures performed after test 1 revealed no significant differences between the 3 groups. The results of the NEO scales and SPI measures vicariously after test 2 are presented in Table 2, and the mean raw scores and standard deviation groupwise measures are presented in Table 3. In test 2, significant group differences occurred at ‘self-reflection’ (*F* (2.22) = 8.56; group 3 vs. 2; *p* < 0.005). From all 3 groups, probands of group 3 achieved the highest increase in accurate self-assessed performance and communicated experience ((*p* < 0.02), multivariate ANOVA (MANOVA) repeated measure, Mauchly test on sphericity: Greenhouse–Geisser 1.00; Huynh–Feldt 1.00).

**Table 2:** Correlations between personality measures, interview ratings after T2, and da Vinci performance

	NEO extraversion	NEO conscientiousness	SPI 2 self-reflection	SPI 2 communication	da Vinci T1 [sec]	da Vinci T2 [sec]
NEO extraversion	1	0.23	0.10	0.09	0.06	0.26
NEO conscientiousness	0.23	1	− 0.14	− 0.04	0.26	0.17
SPI 2 self-reflection	0.10	− 0.14	1	0.79**	−0.40*	− 0.81**
SPI 2 communication	0.09	− 0.04	0.79**	1	−0.44*	−0.60**
da Vinci T1 [sec]	0.06	0.26	− 0.40*	− 0.44*	1	0.57**
da Vinci T2 [sec]	0.26	0.17	− 0.81**	− 0.60**	0.57**	1

*p = 0.05

**p = 0.001

**Table 3:** Mean raw scores and standard deviations of NEO scales and SPI T2 measures groupwise

	NEO extraversion		NEO conscientiousness		NEO openness for experience		SPI 2 self-reflection		SPI 2 communication	
	MW	SD	MW	SD	MW	SD	MW	SD	MW	SD
Group 1 (*n* = 8)	3.40	0.46	3.72	0.50	3.80	0.33	4.21	0.32	3.79	0.38
Group 2 (*n* = 7)	3.46	0.44	3.70	0.29	3.60	0.47	3.62	0.86	3.33	0.79
Group 3 (*n* = 10)	3.49	0.47	3.75	0.38	3.55	0.29	4.23	0.66	3.85	0.82

Significant group differences: SPI 2; self-reflection: *F*(2;22) = 8.088, *p* = 0.002

Thus, the participants in group 2 seemed to be not as capable as those in groups 1 and 3 of communicating and self-evaluating their performance while completing the exercises. The ratings for questions 2, 3 and 5 in self-reflection, especially, were very low (see Fig. 3a).

The training success in group 2 was measured by calculating the difference between the score in the first training trial (test 1) and the score in the last training trial completed by all participants (“end”) for all three training modules (spatial orientation I and II and concentration). Because most of the participants did not follow the instruction to practice 20 times, the last trial (trial 5) from all participants was chosen. The average training gain for group 2 was 24.6% over all three modules and five training trials (see Fig. 3b).

## Discussion

The learning procedure of operational skills of minimally invasive, especially robotic, surgery is currently primarily based on practical training and includes video displays and simulators in classic surgical training on the patient; the video displays and simulators demonstrate (in addition to indications) individual steps of the procedure and train movement patterns.

For several years, various systems have been used for training on the da Vinci operating system, which can be differentiated according to their orientation as follows: web-based robotic surgery programs, on-site training programs and surgical robotic training simulators. The use of the robotic surgery console for training exercises is the major benefit of this system, which enables training with the original robotic console [17–20].

The provided virtual reality (VR) environment enables novice robotic surgeons to practice surgical skills without compromising patient safety; consequently, such simulation training has become an established and reliable component in surgical training on the da Vinci operating system [21]. Generally, complex procedures of robotic-assisted surgery (RAS) based on different motor skills, technical knowledge, cognitive skills, and psychomotoric skills are mandatory [22–24].

Cognitive training is a well-documented and diversified training approach used for complex systems, such as space, aviation and sports. Cognitive training describes the motor system as a part of cognitive network that includes various psychological activities. Cognitive training is also a documented approach to surgical training, although, on average, this approach is less used. In complicated tasks, such as aviation and surgery, the addition of multiple factors (patient condition, disease severity, operating room (OR) environment, complexity of the procedure, etc.) is invariably associated with increased cognitive load, which subsequently influences learning [11, 22, 23, 25, 26]. The cognitive load is defined as the information processed by the working memory, which is limited in its capacity to process information [27]. Continuous practice during learning maximizes the recruitment of working memory [28, 29]. The results of Harbin et al., who were able to demonstrate a positive association between the consumption or use of video games and performance on the da Vinci simulator, accord with these findings [30]. In particular, there was a positive effect on completing certain tasks on the simulator if the gaming experience was more recent. In addition to an immediate or temporally correlated effect, cognitive training also seems to have positive long-term effects on the speed and logic of information processing; additionally, these effects persist for 10 years and is demonstrable [31, 32]. However, the results of these studies do not make it clear which exercises and training procedures are particularly effective in significantly and efficiently increasing surgical skills [24].

In our pilot study, we used modified established da Vinci training modules and a web-based cognitive training focusing on space orientation. The results of the two different training groups show a positive training effect for the da Vinci group, while a deterioration in performance was shown in group 2 after cognitive training. As expected, the control group showed a comparable result in Tests 1 and 2. On closer inspection, the planned training sessions in group 2 have been demonstrably not carried out. Furthermore, at a significantly lower starting level of group 2 (test 1), a random negative selection is to be assumed; this negative selection cannot be compensated for with a small group size and the largest proportion loss of the subject.

On the one hand, it could be assumed that cognitive training does not have a positive effect in this context. On the other hand, the positive correlation between space orientation and motor skills is a proven fact, which is used in the selection and training of astronauts by the European Space Agency (ESA). From this, it would be necessary to carry out training sessions in a correct and suitable form, possibly extended by a motoric component. However, the intensity and form of the cognitive training must be viewed critically; this training was implemented in a significantly reduced manner compared to the necessary measure. Lack of monitoring, unlike in group I, emphasizes this option.

A further aspect of this study, with regard to the optimization of surgical training on the da Vinci surgical robot, is to consider whether there are certain manual skills or personal requirements for successfully training or acquiring surgical skills. In general, there is a recognized connection between personality traits and performance gains and long-term training success: high levels of certain personality traits, such as conscientiousness, extraversion or emotional stability, positively affect learning success and performance; conscientiousness is considered a valid predictor of training performance and correlates with goal setting and preparation quality [14, 15, 36]. In the time interval used in this study, there seems to be a possible short-term effect of cognitive training; we should question this finding. The time interval used in this study may be another factor in the lack of improved performance after cognitive training.

This factor must be considered to design more effective training that replaces or supplements traditional surgical training, which is time consuming and cost intensive; using recent aspects and further features, we propose a model for objective cognitive skills assessment through training [37, 38].

### Limitations

There are different limitations and gaps in our research: The most important deficiency (shortcoming) is the low power and small sample size of different training groups. However, these factors were deliberately accepted as a designed pilot study. A further aspect is the loss of probands in the follow-up, especially in group 2, because they did not follow the instructions to practice exercises repeatedly (20 times). A much tighter controlled training program could prevent this.

Therefore, it is difficult to assess the efficacy and the value of cognitive training in robotic-assisted surgery. For this complicated task, more detailed cognitive features should be used to consider crucial skills, such as stress management, anticipating surprise, situation awareness, aiming period, decision-making, rapid response, managing fear and fatigue. We postulate that modified training models for supporting and improving the visomotoric aspects of cognitive features are necessary. Another interesting aspect in terms of the effectiveness of cognitive training could be the evaluation of the combination of cognitive training and direct training on the da Vinci System.

Former analysis of the DLR test training proves the necessity of a high number of training trials to stabilize the performance gain [16]. Considering that there was no difference in the basic evaluation of the personality characteristics between the 3 groups, the better control of the cognitive training of group 2 seems to be important. Therefore, the results from group 2 should not be interpreted as a low outcome of cognitive training.

## Conclusions

Efficacy of cognitive training in the training of highly complex and difficult procedures, such as RAS, is a proven and accepted fact and is applied in complex environments and organizations like aerospace and sport. In our study we could not prove the obvious assumption that there is also a positive training effect when learning surgical skills. Further investigation with a larger number of subjects on the one hand and a higher numbers of training trials on the other hand should be performed. Furthermore, the combination of cognitive training and direct training on the da Vinci System could be an interesting question.
